# Foundation models for electrocardiogram interpretation: clinical implications

**DOI:** 10.1093/eurheartj/ehaf1119

**Published:** 2026-01-22

**Authors:** Alexis Nolin-Lapalme, Achille Sowa, Jacques Delfrate, Olivier Tastet, Denis Corbin, Merve Kulbay, Derman Ozdemir, Marie-Jeanne Noël, François-Christophe Marois-Blanchet, François Harvey, Surbhi Sharma, Minhaj Ansari, I Min Chiu, Valentina D’souza, Sam F Friedman, Michaël Chassé, Brian J Potter, Jonathan Afilalo, Pierre Adil Elias, Gilbert Jabbour, Mourad Bahani, Marie-Pierre Dubé, Patrick M Boyle, Neal A Chatterjee, Joshua Barrios, Geoffrey H Tison, David Ouyang, Mahnaz Maddah, Shaan Khurshid, Julia Cadrin-Tourigny, Rafik Tadros, Julie Hussin, Robert Avram

**Affiliations:** Department of Biochemistry and Molecular Medicine, Faculty of Medicine, University of Montreal, Montreal, Quebec, Canada H3C 3J7; Montreal Heart Institute, 5000 Rue Bélanger, Montreal, Quebec, Canada H1T 1C8; Mila—Québec AI Institute, Montreal, Quebec, Canada H2S 3H1; Heartwise (Heartwise.ai), Montreal Heart Institute, 5000 Rue Bélanger, Montreal, Quebec, Canada H1T 1C8; Department of Biochemistry and Molecular Medicine, Faculty of Medicine, University of Montreal, Montreal, Quebec, Canada H3C 3J7; Montreal Heart Institute, 5000 Rue Bélanger, Montreal, Quebec, Canada H1T 1C8; Heartwise (Heartwise.ai), Montreal Heart Institute, 5000 Rue Bélanger, Montreal, Quebec, Canada H1T 1C8; Montreal Heart Institute, 5000 Rue Bélanger, Montreal, Quebec, Canada H1T 1C8; Heartwise (Heartwise.ai), Montreal Heart Institute, 5000 Rue Bélanger, Montreal, Quebec, Canada H1T 1C8; Montreal Heart Institute, 5000 Rue Bélanger, Montreal, Quebec, Canada H1T 1C8; Montreal Heart Institute, 5000 Rue Bélanger, Montreal, Quebec, Canada H1T 1C8; Montreal Heart Institute, 5000 Rue Bélanger, Montreal, Quebec, Canada H1T 1C8; Department of Ophthalmology & Visual Sciences, McGill University, Montreal, QC H4A 3S5, Canada; Montreal Heart Institute, 5000 Rue Bélanger, Montreal, Quebec, Canada H1T 1C8; Department of Internal Medicine/Critical Care, Eastern New Mexico Medical Center, Roswell, NM, USA; Montreal Heart Institute, 5000 Rue Bélanger, Montreal, Quebec, Canada H1T 1C8; Center for the Integration and Analysis of Medical Data (CITADEL), Centre Hospitalier de L'Université de Montréal (CHUM), Montreal, Quebec, Canada H2X 0A9; Center for the Integration and Analysis of Medical Data (CITADEL), Centre Hospitalier de L'Université de Montréal (CHUM), Montreal, Quebec, Canada H2X 0A9; Electrophysiology Section, Division of Cardiology, University of Washington, Seattle, WA, USA; Division of Cardiology, Department of Medicine, University of California-San Francisco, San Francisco, CA, USA; Department of Cardiology, Smidt Heart Institute, Cedars-Sinai Medical Center, Los Angeles, CA, USA; Data Sciences Platform, The Broad Institute of MIT and Harvard, Cambridge, MA, USA; Data Sciences Platform, The Broad Institute of MIT and Harvard, Cambridge, MA, USA; Center for the Integration and Analysis of Medical Data (CITADEL), Centre Hospitalier de L'Université de Montréal (CHUM), Montreal, Quebec, Canada H2X 0A9; Cardiovascular Center & Research Center, Centre Hospitalier de L'Université de Montréal (CHUM), Montreal, Quebec, Canada; Division of Cardiology and Centre for Clinical Epidemiology, Jewish General Hospital, McGill University, Montreal, Quebec, Canada; Department of Biomedical Informatics, Columbia University Irving Medical Center, New York, NY, USA; Montreal Heart Institute, 5000 Rue Bélanger, Montreal, Quebec, Canada H1T 1C8; Montreal Heart Institute, 5000 Rue Bélanger, Montreal, Quebec, Canada H1T 1C8; Montreal Heart Institute, 5000 Rue Bélanger, Montreal, Quebec, Canada H1T 1C8; Department of Medicine, Faculty of Medicine, Université de Montréal, Montreal, Quebec, Canada; Electrophysiology Section, Division of Cardiology, University of Washington, Seattle, WA, USA; Electrophysiology Section, Division of Cardiology, University of Washington, Seattle, WA, USA; Division of Cardiology, Department of Medicine, University of California-San Francisco, San Francisco, CA, USA; Division of Cardiology, Department of Medicine, University of California-San Francisco, San Francisco, CA, USA; Department of Cardiology, Smidt Heart Institute, Cedars-Sinai Medical Center, Los Angeles, CA, USA; Division of Research, Kaiser Permanente, Northern California, Pleasanton, CA, USA; Data Sciences Platform, The Broad Institute of MIT and Harvard, Cambridge, MA, USA; Cardiovascular Disease Initiative, Broad Institute of Harvard University and MIT, Cambridge, MA, USA; Cardiovascular Research Center, Massachusetts General Hospital, Boston, MA, USA; Telemachus and Irene Demoulas Family Foundation Center for Cardiac Arrhythmias, Massachusetts General Hospital, Boston, MA, USA; Montreal Heart Institute, 5000 Rue Bélanger, Montreal, Quebec, Canada H1T 1C8; Department of Medicine, Faculty of Medicine, Université de Montréal, Montreal, Quebec, Canada; Montreal Heart Institute, 5000 Rue Bélanger, Montreal, Quebec, Canada H1T 1C8; Department of Medicine, Faculty of Medicine, Université de Montréal, Montreal, Quebec, Canada; Department of Biochemistry and Molecular Medicine, Faculty of Medicine, University of Montreal, Montreal, Quebec, Canada H3C 3J7; Montreal Heart Institute, 5000 Rue Bélanger, Montreal, Quebec, Canada H1T 1C8; Heartwise (Heartwise.ai), Montreal Heart Institute, 5000 Rue Bélanger, Montreal, Quebec, Canada H1T 1C8; Department of Medicine, Faculty of Medicine, Université de Montréal, Montreal, Quebec, Canada; Montreal Heart Institute, 5000 Rue Bélanger, Montreal, Quebec, Canada H1T 1C8; Department of Ophthalmology & Visual Sciences, McGill University, Montreal, QC H4A 3S5, Canada; Department of Medicine, Faculty of Medicine, Université de Montréal, Montreal, Quebec, Canada

**Keywords:** Electrocardiogram, Artificial intelligence, Foundation model, Fairness, Privacy, Generalizability, CLSA, UK Biobank

## Abstract

**Background and Aims:**

The 12-lead electrocardiogram (ECG) remains a cornerstone of cardiac diagnostics, yet existing artificial intelligence (AI) solutions for automated interpretation often lack generalizability, remain closed source, and are primarily trained using supervised learning (SL), which requires extensive labelled datasets and may limit adaptability across diverse clinical settings. Self-supervised learning (SSL) can potentially overcome these limitations by learning robust representations from unlabelled data. To address these challenges, this study developed and compared two open-source foundational ECG models: DeepECG-SL, a supervised multilabel ECG model, and DeepECG-SSL, a self-supervised model.

**Methods:**

Both models were trained on over 1 million ECGs using a standardized preprocessing pipeline and automated free-text extraction from ECG reports to predict 77 cardiac conditions. DeepECG-SSL leveraged unlabelled data through self-supervised contrastive learning and masked lead modelling before fine-tuning for downstream tasks, while DeepECG-SL was trained directly on labelled diagnostic data in an end-to-end fashion. Performance was evaluated across seven private, multilingual healthcare systems and four public ECG repositories, with assessment of fairness by age and sex, and investigation of privacy vulnerabilities as well as memory and compute requirements.

**Results:**

DeepECG-SSL achieved micro-averaged area under the receiver operating characteristic curves (AUROCs) across all 77 cardiac conditions for ECG interpretation of 0.990 [95% confidence interval (CI): 0.990, 0.990] on the internal dataset (MHI-ds), 0.981 (95% CI: 0.981, 0.981) on external public datasets (UKB, CLSA, MIMIC-IV and PTB), and 0.983 (95% CI: 0.983, 0.983) on external private datasets (UW, UCSF, JGH, NYP, MGH, CSH and CHUM), while DeepECG-SL demonstrated AUROCs of 0.992 (95% CI: 0.992, 0.992), 0.980 (95% CI: 0.980, 0.980), and 0.983 (95% CI: 0.983, 0.984), respectively. Fairness analyses revealed minimal disparities (true-positive rate and false-positive rate difference <0.1) across age and sex groups for both models. DeepECG-SSL demonstrated superior performance on limited-data digital biomarker tasks, with the largest improvements in long QT syndrome (LQTS) genotype classification (AUROC 0.931 vs 0.850, *P* = .026, *n* = 127 ECGs) and 5 year atrial fibrillation risk prediction (AUROC 0.742 vs 0.734, *P* < 0.001, *n* = 132 050 ECGs), while achieving superior performance in left ventricular ejection fraction ≤40% classification (AUROC 0.926 vs 0.917, *P* < 0.001, *n* = 25 252 ECGs) and comparable performance in LQTS detection (AUROC 0.767 vs 0.735, *P* = 0.117, *n* = 934 ECGs).

**Conclusions:**

This study establishes SSL as a promising paradigm for ECG analysis, particularly in settings with limited annotated data, enhancing accessibility, generalizability, and fairness in AI-driven cardiac diagnostics. By releasing model weights, preprocessing tools, and validation code, this work aims to support robust, data-efficient AI diagnostics across diverse clinical environments and questions.

## Structured Graphical Abstract

**Graphical Abstract:**
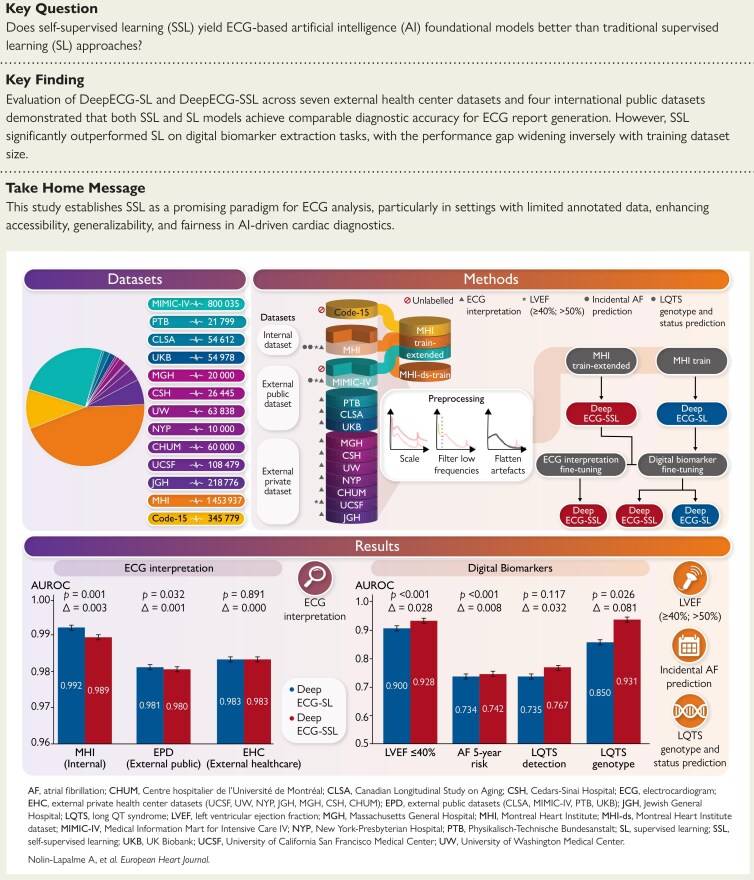
This study compared DeepECG-SSL and DeepECG-SL models trained on over 1 million ECGs and evaluated on 13 datasets across North America and Europe. The SSL approach leveraged unlabelled ECGs from Code-15 and MIMIC-IV for pretraining, while SL used labelled ECGs from the MHI (a tertiary care centre). Both models underwent fine-tuning for ECG interpretation and digital biomarker extraction tasks, including left ventricular ejection fraction (LVEF) prediction, 5 year atrial fibrillation (AF) risk, and long QT syndrome (LQTS) detection and genotype classification. External validation encompassed four public datasets (CLSA, MIMIC-IV, PTB, UKB) and seven private healthcare centre datasets. Results demonstrated equivalent performance for ECG interpretation (AUROC 0.980-0.992), while SSL significantly outperformed SL for digital biomarker tasks, particularly LVEF ≤40% prediction (AUROC 0.928 vs 0.900, *P* < 0.001) and LQTS genotype classification (AUROC 0.931 vs 0.850, *P* = 0.026). The performance gap between SSL and SL widened as training dataset size decreased, supporting SSL as a robust paradigm for ECG analysis in data-limited settings. This study compared DeepECG-SSL and DeepECG-SL models trained on over 2.5 million ECGs from 13 datasets across North America and Europe. The SSL approach leveraged unlabelled ECGs from Code-15 and MIMIC-IV for pretraining, while SL used labelled ECGs from the Montreal Heart Institute (MHI). Both models underwent fine-tuning for ECG interpretation and digital biomarker extraction tasks, including left ventricular ejection fraction (LVEF) prediction, 5 year atrial fibrillation (AF) risk, and long QT syndrome (LQTS) detection and genotype classification. External validation encompassed four public datasets (CLSA, MIMIC-IV, PTB, UKB) and seven private healthcare centre datasets. Results demonstrated equivalent performance for ECG interpretation (AUROC 0.980-0.992), while SSL significantly outperformed SL for digital biomarker tasks, particularly LVEF ≤40% prediction (AUROC 0.928 vs 0.900, *P* < 0.001) and LQTS genotype classification (AUROC 0.931 vs 0.850, *P* = 0.026). The performance gap between SSL and SL widened as training dataset size decreased, supporting SSL as a robust paradigm for ECG analysis in data-limited settings.

**See the editorial comment for this article ‘Artificial intelligence-enhanced electrocardiograms: building on solid foundations’, by F.S. Ng**  ***et al.*****, https://doi.org/10.1093/eurheartj/ehaf1008.**

## Introduction

The 12-lead electrocardiogram (ECG) is a fundamental cardiovascular diagnostic tool, with over 300 million performed annually.^1^ Artificial intelligence (AI) algorithms now rival or surpass clinicians in detecting arrhythmias and ischaemia,^2,3^ and emerging ECG-AI applications turn the ECG into a digital biomarker for conditions beyond its traditional scope, including left ventricular dysfunction,^4^ pulmonary hypertension,^5^ and inherited arrhythmia genotypes such as long QT syndrome (LQTS).^6^

State-of-the-art ECG-AI predominantly uses supervised learning (SL), which depends on large, task-specific labelled datasets.^7^ In contrast, self-supervised learning (SSL) leverages abundant unlabelled recordings to learn internal representations^8^ that can be fine-tuned on much smaller labelled cohorts, often achieving superior performance and broader generalizability.^9–11^ Despite these advantages, few studies directly compare SL and SSL for ECG-AI, and only 14% of medical AI research shares code or data publicly, hindering reproducibility and collaboration.^12,13^

Here we introduce two open-source ECG foundation models for multi-task generalizability: DeepECG-SL, trained by multilabel SL on 77 ECG diagnoses (see Supplementary data online, *Table S2*), and DeepECG-SSL, pre-trained on 1.9 million ECGs from three cohorts using contrastive learning and masked-lead modeling^14^ and then fine-tuned on downstream tasks. We evaluate both models on 77-label ECG report classification, transthoracic left ventricular ejection fraction (LVEF) estimation, 5 year incident atrial fibrillation (iAF5) prediction in patients in sinus rhythm without prior atrial fibrillation, and LQTS diagnosis and subtyping. Validation across 11 datasets (seven institutional, four public; total *n* = 881 403 ECGs) assesses predictive accuracy, fairness across sex and age, privacy considerations, and computational efficiency.

## Methods

This retrospective study was approved by the Ethics Board of the Montreal Heart Institute (MHI) for multicentre development and validation (Project #2023-3160), and all research activities were in accordance with the Declaration of Helsinki (2013).^15^ During manuscript preparation, large language models were used to assist with editorial and stylistic revisions. The authors take full responsibility for the scientific integrity, accuracy, and interpretation of the content presented herein.

### Training datasets

The internal dataset, MHI dataset (MHI-ds), contains 1 453 937 12-lead ECGs from 263 143 patients. As a specialized quaternary care hospital, the MHI treats a wide range of cardiovascular conditions, making its dataset ideal for training a foundation model. These ECGs were recorded at the MHI between 11 April 1997, and 10 November 2023, using the MUSE system (General Electric, Boston), and were randomly split into training (MHI-ds-train—70%), validation (MHI-ds-val—10%), and test (MHI-ds-test—20%) partitioned at a patient level, ensuring that no single patient’s ECG appeared in more than one split. Age, sex, and label distributions were carefully balanced across all splits. For DeepECG-SL training, we used only MHI-ds-train with its full 77 diagnostic labels for SL. For DeepECG-SSL training, we created the MHI-train-extended dataset for self-supervised pre-training, which combined MHI-ds-train with two public datasets: the Code-15 dataset (345 779 ECGs from 233 770 patients)^16^ and the unlabelled MIMIC-IV-train, representing 70% (558 464 ECGs from 112 902 patients) of the full MIMIC-IV dataset^17^ (*Structured Graphical Abstract*). During SSL pre-training, all labels were ignored to enable learning from the raw ECG signals alone. After pre-training, DeepECG-SSL was fine-tuned using the labelled MHI-ds-train dataset. We applied similar constraints on age, sex, and label distribution as well as patient partitioning when splitting MIMIC-IV into MIMIC-IV-train (70%) and MIMIC-IV-test (30%).

### External validation

To evaluate our models beyond the scope of the MIMIC-IV-test and the MHI-ds-test sets, we defined all publicly available ECG datasets or biobanks as ‘External Public Datasets’ (EPD). These include the Canadian Longitudinal Study on Aging^18^ (CLSA), a stratified sample of 29 427 Canadian adults aged 45–85 years, from which we used baseline (2011) ECGs (*n* = 29 427) and first follow-up ECGs (2015–2018; *n* = 25 331; total; *n* = 54 612). We also incorporated data from the UK Biobank (UKB)^19^ (Project #20168) collected during the first imaging visit in 2014 and a follow-up in 2019 (*n* = 54 978 ECGs), as well as the Physikalisch-Technische Bundesanstalt^20^ (PTB) dataset with PTB-XL annotations (*n* = 21 799) (Additional details on each cohort are provided in Supplementary data online, *Table S35*).

To further assess clinical performance and generalizability, we validated both models at multiple healthcare institutions, collectively designated as the ‘External Health Center (EHC) datasets. It comprises ECGs from the following healthcare centres: the University of California, San Francisco (UCSF; 108 479 ECGs), Massachusetts General Hospital (MGH; 20 000 ECGs), Cedars-Sinai Hospital (CSH; 26 445 ECGs), Jewish General Hospital (JGH; 218 776 ECGs), the University of Washington Medical Center (UW; 63 838 ECGs), New York-Presbyterian Hospital (NYP; 10 000 ECGs), the Centre Hospitalier de l’Université de Montréal (CHUM; 60 000 ECGs). Each institution’s data were curated according to the same criteria used for the training set and the external public datasets, ensuring uniform preprocessing and evaluation.

### ECG signal preprocessing

To enable our models to work across all hospitals and datasets regardless of equipment, we developed an automated three-step preprocessing pipeline that cleans ECGs while preserving diagnostic information:

1. **High-pass filtering.** We computed each ECG’s Fast Fourier Transform to compare energy below 1 Hz with the 1–30 Hz diagnostic band^21,22^; if the low-frequency component exceeded the higher-frequency band by an order of magnitude, we applied a zero-phase 1 Hz high-pass filter.
2. **Artefact suppression.** Narrowband artefacts (such as A/C current-related 50/60 Hz interference^23^) were detected when their local amplitude exceeds two standard deviations (see Supplementary data online, *Figure S1A*) above the dataset mean, then flattened using a LOESS fit.^24^
3. **Amplitude scaling.** Finally, we rescaled every signal to match MHI-ds’s millivolt range (see Supplementary data online, *Figure S1*).

Applying this pipeline to all non-reference cohorts (with MHI-ds adjusted only to express voltages in millivolts), significantly improved cross-dataset generalization without compromising clinically relevant 1–30 Hz features (see Supplementary data online, *Table S10*). The 1–30 Hz band was selected as it contains the bulk of diagnostically relevant ECG signal while enabling detection of baseline wander (<1 Hz) primarily from motion artefacts.^21,22^ Our pipeline preserves the full 0–150 Hz spectrum but uses the 1–30 Hz band only as a reference for identifying excessive low-frequency noise.

### ECG interpretation task

We mapped free-text ECG reports to 77 diagnostic labels using a multi-step, expert-driven process. Two cardiologists (≥4 years of ECG interpretation experience) independently annotated 10 075 unique diagnostic statements (4200 MHI-ds, 3811 MIMIC-IV, 2064 UKB) using a 77-label framework based on American Heart Association recommendations.^25^ Waveforms were reviewed in standard 12-lead format, any disagreements were resolved by a third cardiologist, and any condition present anywhere in the 10 s recording was labelled at the record level with an inter-rater reliability of Cohen’s κ > 0.80 for all diagnostic labels.

Dataset labelling varied by source: MHI-ds and MIMIC-IV contained full diagnostic reports enabling extraction of all 77 labels through direct dictionary mapping. Code-15 provided only 6 labels (<8% of our framework), so it was treated as unlabelled for SSL pre-training only. PTB included manual annotations requiring dictionary conversion. UKB underwent direct dictionary mapping from its existing labels.

To train a multilingual classifier, since CHUM and MHI-ds data contained French diagnoses, we expanded these annotations into 160 129 paragraphs, augmented with 150 000 shuffled paragraph pairs using LLAMA 3.1 70B^26^ and bidirectional French-English translation, yielding 640 518 paragraph-label pairs. This corpus was split 70/10/20 to train a BERT-based^27^ model for robust mapping of clinical statements to our 77-category schema. This BERT classifier was then applied to label CLSA and all EHC. During SSL pre-training, MHI-train-extended combined three unlabelled datasets: MHI-ds-train, Code-15, and MIMIC-IV-train. Labels were utilized only during supervised fine-tuning for downstream tasks. Further details appear in Supplementary data online, *Tables S3* and *S44*, Supplementary Methodology  *S1* and Supplementary data online, *Figure S13*.

### Algorithm development and evaluation

#### DeepECG-SL

DeepECG-SL was trained on MHI-ds-train for ECG interpretation across 77 diagnoses, through an extensive sweep comparing different architectures, including families of transformers,^28^ convolutional neural networks (CNN), and mamba.^29^ The training also explored various data augmentation strategies, training environment parameters, initialization schemes, regularization techniques, and preprocessing strategies. To identify the optimal SL architecture, we utilized the Weights and Biases platform,^30^ leveraging a Bayesian optimizer to maximize the macro-averaged area under the precision-recall curve (AUPRC) and ensure hyper-parameter optimization (see Supplementary data online, *Table S7*, Supplementary Methods  *S3*). Initialized with the weights optimized for ECG interpretation of our best performing model, DeepECG-SL was then fine-tuned for each digital biomarker prediction task.

#### DeepECG-SSL

We trained DeepECG-SSL in two stages, first self-supervised pre-training on the MHI-train-extended ECGs and then supervised fine-tuning on MHI-ds-train for ECG interpretation and downstream biomarker tasks (see Supplementary data online, Methods  *S4*). For pre-training we adopted the framework of Oh *et al.*^14^ which merges Wav2Vec^31^ for learning local features with Contrastive Multi-Segment Coding^32^ for global signal representations (see Supplementary data online, *Table S9*). In practice, each ECG is split into two consecutive segments, a random subset of leads is masked, and both masked and unmasked segments are passed through a convolutional encoder whose features are quantized, and masked tokens are replaced. A transformer encoder then produces contextual embeddings, and two contrastive losses guide learning: one aligns each token’s context with its quantized version within the same segment, and the other draws together global averages of segments from the same ECG or patient while pushing apart those from other recordings (see Supplementary data online, *Figure S2*). After pre-training, we unfroze all layers and replaced the head with a task-specific classifier to fine-tune on labelled ECG and biomarker endpoints.

Both models were trained using four NVIDIA RTX A6000 graphics processing units (GPUs). The architecture search for DeepECG-SL was conducted in parallel with one GPU for each model family, with each search running for two weeks. DeepECG-SSL was pre-trained on three NVIDIA RTX A6000 GPUs over a period of 14 days.

### Digital biomarker tasks

After establishing performance on standard ECG interpretation, we evaluated both models on emerging biomarker applications with varying data availability: LVEF regression and classification, LQTS detection and type classification, and iAF5 prediction. These tasks were chosen because we had sufficiently large, annotated datasets for each across our external validation sites, and our team has previously published^6,33^ on these clinical outcomes, allowing direct comparison with previous work. These tasks also encompass processes invisible to the human eye, hence the term *biomarker*. Moreover, they span varying levels of supervisory complexity, ranging from binary classification to continuous-value regression, while leveraging datasets of highly heterogeneous sizes. All models were fine-tuned on MHI-ds-derived data, and we ensured that each specialized task used overlapping data with the MHI-ds-train and MHI-ds-test split to prevent any data leakage.

For DeepECG-SL, we started with the model trained for ECG interpretation, while DeepECG-SSL was initialized with its pre-training weights. To fine-tune the model, we unfroze all the layers and adjusted the classifier to have a single scalar output for each biomarker task (binary or regression) (further details in Supplementary Methods  *S5*).

#### LVEF prediction

For LVEF-related tasks, we fine-tuned our models on MHI-ds-train and evaluated them on MHI-ds-test, CSH, NYP, UCSF, UW, JGH, and MIMIC-IV datasets. Each ECG was paired with the closest transthoracic echocardiography report recorded within a time window of −90 to +7 days of ECG recordings. In cases where multiple ECGs matched this criterion for the same patient, only the closest instance was selected. For the regression task, we used the LVEF values, while for classification tasks, we dichotomized LVEF values as <50% and ≤40% to correspond to different severity thresholds for two distinct classification tasks.

#### LQTS identification and subtype classification

For the LQTS tasks, we followed an approach based on Jiang *et al.*^6^ leveraging only MHI-ds. Patients were labelled based on the presence of pathogenic or likely pathogenic variants in the *KCNQ1* or *KCNH2* genes, or a familial LQTS-causing genetic variant, which allowed us to differentiate between LQTS type 1 and type 2. Variants of uncertain significance (VUS) were reviewed by electrophysiologists specializing in inherited arrhythmias and classified as either strong VUS or weak VUS. Patients with strong VUS were labelled as LQTS cases, while those with weak VUS were used as controls for LQTS identification. Positive cases were further subdivided into type 1 and type 2 for subtype classification. This design allowed us to create both an LQTS identification task and a subtype classification task.

#### iAF5 prediction

For iAF5 labelling, we followed the methodology outlined in our previous study.^33^ The model was fine-tuned on the appropriate subset of MHI-ds-train and tested on MHI-ds-test, MIMIC-IV-test, CSH, and UCSF for examples with appropriate labels. ECGs were included if they had valid metadata, had waveform data, and were confirmed to be in sinus rhythm. Additional exclusion criteria included a proximity of less than 30 days to cardiac surgery or the presence of atrial flutter on any ECG belonging to the patients in this dataset.

### Fairness, privacy, and energy consumption analysis

To ensure equitable performance, we assessed algorithmic fairness using the Equalized Odds framework,^34^ which required parity of both the true-positive rate (TPR) and false-positive rate (FPR) across protected groups. In this analysis, we categorized age into three groups: under 55, 55–75, and over 75. Fairness was assessed by evaluating the performance of both models on the ECG interpretation task, where the TPR and FPR were computed for each group defined by age and sex, using micro-averaging across all 77 classes. When comparing fairness with ECGFounder^35^ and ECG-FM^36^ models, this was performed on overlapping labels (see Supplementary data online, *Tables S4* and *S41*).

For privacy evaluation, we tested vulnerability to membership inference attacks (MIA),^37^ a method where an adversary attempts to determine whether a specific patient’s ECG was used to train the model, potentially exposing protected health information. Such attacks pose serious risks for clinical AI deployment, as successful identification could violate patient privacy regulations. We implemented MIA using tenfold cross-validation: for each fold, we created balanced datasets combining training samples in MHI-ds-train (positive class) with test samples (negative class) from five external cohorts. A Random Forest classifier was trained (50 trees, 80% of the dataset) to distinguish training from test samples based on the model’s 77-dimensional output predictions. Attack success was measured by classification accuracy; higher accuracy indicates greater privacy vulnerability. We further analysed which diagnostic predictions were most revealing using feature importance scores and visualized data separation patterns using t-SNE^38^ to understand how dataset-specific characteristics enable re-identification.

Finally, we compared the energy footprint and computational efficiency of DeepECG-SL and DeepECG-SSL. We measured inference time, energy consumption, and CO₂ emissions on both GPU and CPU, alongside model size (see Supplementary Methods  *S6*).

### Explainability

To identify which ECG segments drive each prediction, we applied Local Interpretable Model-Agnostic Explanations (LIME)^39^ and Gradient-weighted Class Activation Mapping (Grad-CAM)^40^ to produce saliency maps over time windows and leads. LIME generates local perturbations and measures their impact on the predicted probability, attributing positive values to segments that increase it. Grad-CAM computes the gradient of the target score with respect to feature activations, yielding an activation map of the most critical temporal segments and leads. In our CNN-based DeepECG-SL, Grad-CAM was applied to the final convolutional layer; in the transformer-based model, last-attention token embeddings were reshaped into a continuous time series and Grad-CAM was computed on the final self-attention block. These approaches were also used on ECGFounder^35^ and ECG-FM^36^ models. Each label in our multilabel framework was analysed independently (see Supplementary Methods  *S7*).

### Evaluation metrics and statistical analysis

We measured discrimination by micro-averaged AUROC and AUPRC, sensitivity, and specificity. For all metrics in this study, we derived 95% CIs via 1000 bootstrap iterations sampling 70% of the data each time. An ‘Overall’ score, which concatenates all label predictions and ground truths, served as our micro-average; when CIs did not overlap, we reported ΔSSL–SL. AUROC comparisons used a two-sided DeLong test^41^ while other metrics were compared using a Wilcoxon test (*P* < .05). To quantify pre-training benefits, we trained models end-to-end from random initialization on the same digital-biomarker tasks and compared their performance to DeepECG-SL, DeepECG-SSL, as well as our final models to recent foundation models ECGFounder^35^ and ECG-FM^36^ on the 77-label ECG classification, using a conversion table (see Supplementary data online, *Table S41*), and iAF5 prediction tasks. Calibration was evaluated using the weighted Brier score presented by Zhu *et al.*^42^ while model comparison was done using the net reclassification index (NRI)^43^ between DeepECG-SL, DeepECG-SSL, ECGFounder, and ECG-FM. As this analysis requires the same number of labels in the comparison, this was performed on overlapping labels (see Supplementary data online, *Table S4*). For LVEF regression, we report mean absolute error (MAE). We optimized each label’s decision threshold to balance sensitivity and specificity on MHI-ds-val and then applied those thresholds to all cohorts. External datasets were grouped into external public datasets (EPD: MIMIC-IV, PTB, CLSA, UKB) and External Healthcare Centers (EHC: UW, UCSF, MGH, CSH, NYP, JGH, CHUM), with dataset-level metrics in Supplementary data online, *Tables S11*–*S24*. We also applied the same explainability approaches on ECGFounder and ECG-FM models (see Supplementary Methods  *S7*).

Finally, fairness was measured via equalized odds, requiring similar true-positive rates [TPR = TP / (TP + FN)] and false-positive rates [FPR = FP / (FP + TN)] across demographic groups, by computing TPR and FPR at the optimized thresholds and reporting ΔTPR and ΔFPR, with smaller values indicating better parity.

## Results

DeepECG-SL and DeepECG-SSL represent two distinct approaches to ECG-AI analysis. DeepECG-SL is trained on 1 017 720 ECGs across 184 210 patients. The models were developed using the MHI-ds-train, which maintains balanced demographics (mean age 60.40 ± 0.08 years with an 11.72% male bias), with DeepECG-SSL additionally leveraging ECG signal data from Code-15 and the MIMIC-IV-train datasets in the MHI-train-extended dataset for pre-training (*Table 1*, Supplementary data online, *Table S35*).

**Table 1:** Overall demographics of the training set used in this study

Dataset	MHI-ds-train	MIMIC-IV-train	CODE-15
Number of ECG	1 017 720	558 464	345 779
Mean exam per patient	5.52 (95% CI: 5.49–5.56)	4.95 (95% CI: 4.90–4.99)	1.48 (95% CI: 1.48–1.48)
Mean age per patient	60.40 (95% CI: 60.33–60.48)	58.18 (95% CI: 58.07–58.30)	51.00 (95% CI: 50.92–51.08)
Number of patients	184 210	112 902	233 770
Gender distribution			
Male	102 903 (55.86%)	53 224 (47.14%)	94 807 (40.56%)
Female	81 307 (44.14%)	58 474 (51.79%)	138 963 (59.44%)
Unknown	*-*	1 204 (1.07%)	*-*

MHI-ds-train was used to train DeepECG-SL, and in combination with MIMIC-IV-train and Code-15 for DeepECG-SSL. To calculate the age distribution, the age was first averaged across all patients and then averaged throughout the dataset. The gender distribution is reported at the patient level.

MHI, Montreal Heart Institute Dataset; MIMIC-IV, Medical Information Mart for Intensive Care IV Dataset; ECG, electrocardiogram.

### ECG interpretation task

Our BERT annotation model achieved high classification performance, with the AUROC >0.999 across all classes (except *U* wave, AUROC = 0.982) on the test set, which included 20% of the PTB, MIMIC-IV-test, and MHI-ds datasets. These predictions served as accurate ground truth for the EHC and CLSA datasets (see Supplementary data online, *Table S3*), while PTB relied on its existing manual annotations and MIMIC-IV and UKB used direct dictionary mapping. Our preprocessing pipeline (frequency–domain cleaning, 1 Hz high-pass filtering, and artefact removal) dramatically improved cross-dataset performance, increasing AUROC by up to 0.251 (MHI-ds: 0.741 to 0.992, CLSA: 0.828 to 0.995, UKB: 0.813 to 0.988), while maintaining strong performance on frequency-matched datasets (PTB, MIMIC-IV; Supplementary data online, *Table S10*).

**Figure 1:**
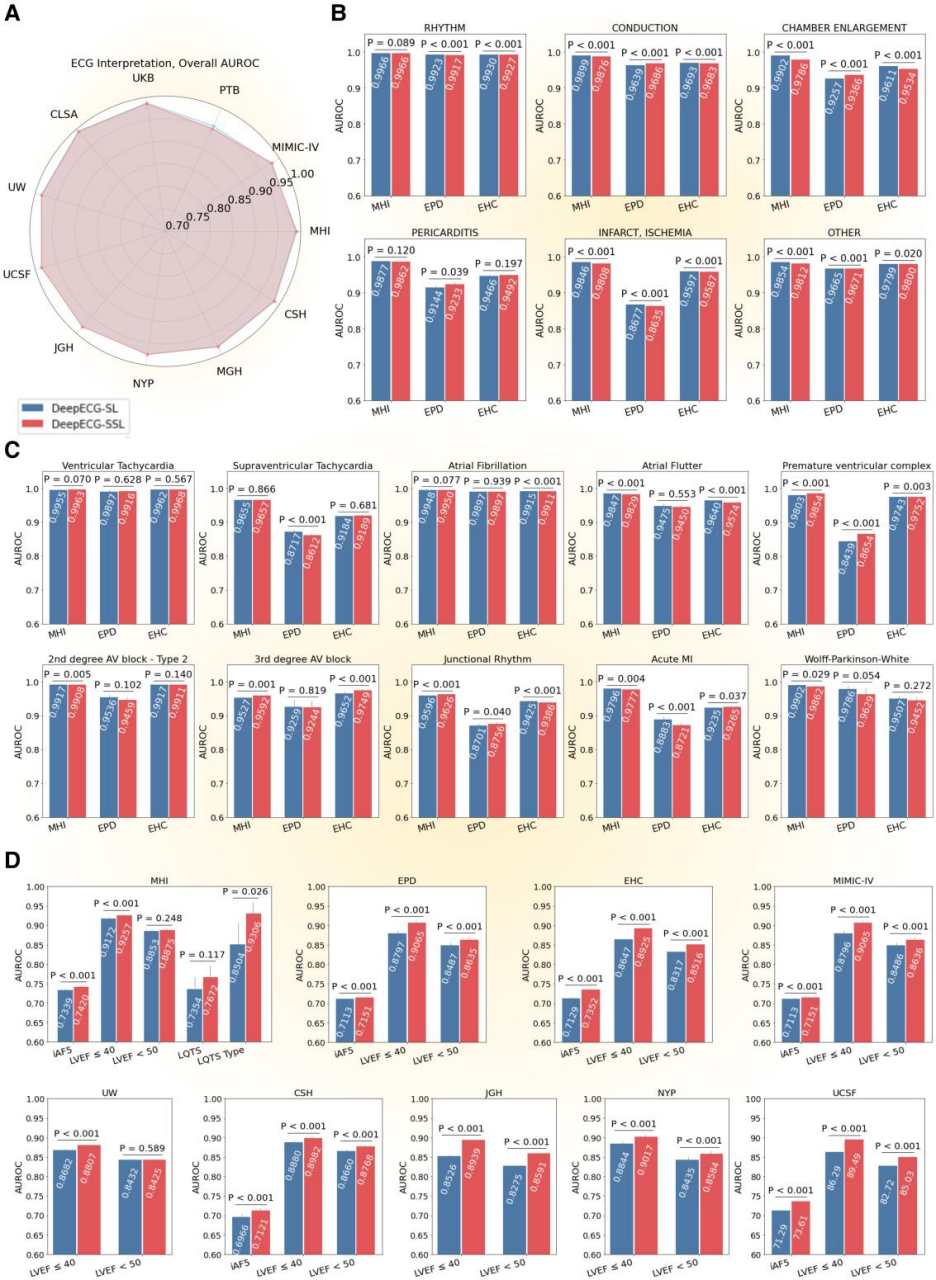
An overview of DeepECG-SL and DeepECG-SSL performances. We report overall AUROC and *P*-value computed using the DeLong test. (*A*) Overall AUROC of ECG interpretation across all datasets. (*B*) Global AUROC of ECG interpretation categories on MHI-ds, EPD, and EHC. (*C*) Label-specific AUROC of ECG on MHI-ds, EPD and EHC. (*D*) AUROC of digital biomarkers across datasets. iAF5 is at ECG level. CSH (Cedars-Sinai Hospital Dataset; CLSA, Canadian Longitudinal Study on Aging Dataset; ECG, electrocardiogram; EHC, External Health Center Dataset; EPD, external public dataset; JGH, Jewish General Hospital Dataset; LQTS, long QT syndrome; LVEF, left ventricular ejection fraction; MGH, Massachusetts General Hospital Dataset; MHI, Montreal Heart Institute Dataset; MIMIC-IV, Medical Information Mart for Intensive Care IV Dataset; NYP, New York-Presbyterian Hospital Dataset; PTB, Physikalisch-Technische Bundesanstalt Dataset; UKB, UK Biobank Dataset; UCSF, University of California San Francisco Medical Center Dataset; UW, University of Washington Medical Center; CHUM, Centre hospitalier de l’Université de Montréal; and WCR, Wave2Vec + Contrastive Multi-segment Coding + Random Lead Masking

After an extensive sweep (670 different training runs), DeepECG-SL achieved the highest performance for ECG interpretation on MHI-ds-val using a modified EfficientNet-V2-S architecture^44^ optimized for one-dimensional signals (see Supplementary data online, *Tables S7* and *S34*). DeepECG-SSL employed a two-stage approach, with self-supervised pre-training for 14 days and fine-tuning for downstream tasks using the architecture described above, such as ECG interpretation. Both models achieved high performance across internal and external datasets (*Figure 1*, Supplementary data online, *Tables S11–S24*). On MHI-ds-test (*n* = 287 039), DeepECG-SL achieved AUROC 0.992, marginally outperforming DeepECG-SSL (0.990). Performance remained robust on external public datasets (EPD; *n* = 373 865; AUROC 0.981 vs 0.980) and healthcare centres (EHC; *n* = 507 538; AUROC 0.983 both models).

Major diagnostic categories showed consistent performance: rhythm disorders (AUROC >0.92), conduction abnormalities (>0.96), and chamber enlargement (>0.92), with minimal differences between models (Δ < 0.01). However, myocardial infarct/ischaemia detection showed reduced performance on EPD (AUROC 0.867 SL vs 0.863 SSL) compared with MHI (0.984 vs 0.980) and EHC (0.954 both). SSL improved calibration on external datasets, particularly PTB (Δ −0.013) and MIMIC (Δ −0.013), driven by better rhythm and ischaemia calibration. Detailed metrics are provided in Supplementary data online, *Table S43*.

### Digital biomarker tasks

We evaluated three AI-derived digital biomarkers, representing ECG-based predictions that surpass the scope of traditional electrocardiographic interpretation. These included the following: (i) LQTS detection and subtype classification (*KCNQ1* vs *KCNH2* variants), (ii) left ventricular ejection fraction estimation (classification tasks: LVEF ≤40% and <50% and regression tasks), and (iii) iAF5 risk prediction from sinus rhythm ECGs. These tasks, having varied labelled data availability, served as stringent tests of model generalization to novel tasks.

For digital biomarkers (*Figure 1C*, Supplementary data online, *Tables S25*–*S33*), DeepECG-SSL significantly outperformed DeepECG-SL in predicting iAF5risk (AUROC: 0.742 vs 0.734; Δ = 0.008; *P* < .001) and identifying LVEF ≤40% (0.926 vs 0.917; Δ = 0.009; *P* < .001), while achieving comparable performance in LQTS detection (0.767 vs 0.735, *P* = .117, *n* = 934) on our internal dataset. Across external datasets, DeepECG-SSL maintained consistent superiority for iAF5 and LVEF predictions (all *P* < .001, except LVEF <50% at UW).

Performance divergence correlated with training dataset size: comparable for standard 77-diagnosis interpretation (SL and SSL fine-tuning *n* = 1 017 720), modest difference for LVEF <50 prediction (*n* = 537,742, ΔAUROC = 0.008), but substantial SSL advantage for LQTS genetic subtyping (type 1 vs 2, *n* = 334, ΔAUROC = 0.081, *P* = .026), demonstrating SSL’s superior performance in data-limited scenarios.

To understand how training data volume affects performance, we tested both models using progressively smaller portions of our training dataset. Both performed similarly when using at least 10% of available data (ΔAUROC = 0.00), but DeepECG-SSL showed clear advantages when data were extremely limited, as presented with 1% of training data (ΔAUROC = 0.09), highlighting its potential value for rare diseases or novel clinical applications (see Supplementary data online, *Figure S4*).

We also compared our pre-trained models against versions trained from scratch (‘end-to-end’ or E2E models) without any prior learning (see Supplementary data online, *Table S35*). These E2E models, which start with random weights rather than pre-trained knowledge, performed poorly in data-limited scenarios; for LQTS subtyping, the pre-trained models achieved AUROC of 0.875 (95% CI: 0.849–0.906) vs only 0.602 (95% CI: 0.533–0.672) for E2E versions. However, for data-rich tasks like LVEF <50% prediction, E2E models achieved respectable performance (0.909 vs 0.839), with DeepECG-SL-E2E surprisingly outperforming its SSL counterpart, suggesting that pre-training benefits are most pronounced when labelled data are scarce (see Supplementary data online, *Table S37*).

### Benchmarking against state-of-the-art models

We evaluated both ECG-FM and ECGFounder for ECG interpretation using the UKB dataset. Out of the 150 labels predicted by ECGFounder, 47 overlapped with the 77 labels predicted by our models. Similarly, 14 of the 17 labels predicted by ECG-FM intersected with our label set. The label mappings used for alignment are detailed in Supplementary data online, *Table S41*.

A direct comparison across 14 shared diagnostic classes demonstrated the modest but consistent superiority of DeepECG-SSL and DeepECG-SL over all other models tested on the UKB, CLSA, and PTB datasets (see Supplementary data online, *Tables S38*–*S40*). For example, on the UKB dataset, DeepECG-SSL and DeepECG-SL achieved an overall AUROC of 0.997, slightly outperforming ECGFounder (0.98) and ECG-FM (0.969). The most pronounced performance gains were observed in CONDUCTION (Δ = 0.045). For ECG interpretation, upsampling the signals to 500 Hz led to improved performance for both ECGFounder and ECG-FM. We fine-tuned ECGFounder and ECG-FM on the MHI-ds-train dataset for the iAF5 digital biomarker (see Supplementary data online, *Table S42*). All four models performed comparably on MHI-ds-test (AUROC 0.73–0.74), but DeepECG-SSL showed markedly stronger external generalization on MIMIC-IV-test (AUROC 0.715 vs 0.711, 0.62, and 0.573). Across all EPD datasets, DeepECG-SSL and DeepECG-SL consistently delivered higher NRI than ECGFounder and ECG-FM (see Supplementary data online, *Figure S15*), with DeepECG-SSL achieving the largest gains on UKB and CLSA (NRI > + 0.87 on shared labels). DeepECG-SL remained strongest on MHI and PTB.

### Fairness and privacy

Both models demonstrated strong fairness across demographics (TPR differences <0.1 and FPR differences <0.02), with DeepECG-SSL showing marginally better balance across age and gender groups (see Supplementary data online, *Figure S14*).

Both models showed strong privacy preservation on internal MHI-ds data (AUROC <0.6) and PTB dataset (AUROC <0.78). However, external datasets with distinct feature distributions, particularly MIMIC-IV and UKB, showed higher re-identification rates (AUROC >0.95), suggesting that despite architecture variation both models remained susceptible to MIA-based attacks (see Supplementary data online, *Table S5*). Analysis of membership inference attack patterns revealed that re-identification success was driven by dataset characteristics rather than model architecture. Datasets where models relied heavily on specific features showed higher re-identification rates, while those with distributed feature importance demonstrated better privacy protection. t-SNE visualization of model outputs confirmed this pattern: greater separation between training and test set representations correlated with higher re-identification rates (see Supplementary data online, *Figure S5*). For instance, in CLSA, DeepECG-SL’s disproportionate weighting of specific features (‘No QRS,’ ‘Regular,’ ‘Monomorph’) led to more distinct clusters and increased re-identification risk.

### Resource usage

DeepECG-SSL demonstrated higher computational demands compared with DeepECG-SL across several metrics. The SSL model is substantially larger (90.37 M vs 1.51 M parameters; 60-fold increase) and requires more operations (14.17 GMAC vs 530.57 MMAC; 27-fold increase). For processing 1000 ECGs on GPU, DeepECG-SSL consumes more energy (0.7463 vs 0.1786 Wh; 318% increase) and produces higher CO₂ emissions (1.774 vs 0.425 mgCO₂). To contextualize, processing 1 million ECGs with DeepECG-SL on CPU produces CO₂ emissions equivalent to driving a car for about 0.79 s at 100 mph, compared with 7.69 s for DeepECG-SSL (see Supplementary data online, *Table S6*).

### Explainability

LIME and Grad-CAM approaches provided qualitative insight, but not a comprehensive interpretability assessment for the entire dataset.^45^ In the examples we reviewed, both DeepECG-SL and DeepECG-SSL tend to highlight features that align with known clinical markers with Grad-CAM generally highlighting more clinically valuable sections such as extra beats or abnormal beat timing in the atrial fibrillation example or a clear focus on ST elevation in the acute infarction example. Moreover, DeepECG-SSL’s heatmaps are generally more focused, suggesting concentration on fewer, potentially more discriminative segments, whereas DeepECG-SL exhibits broader attributions particularly on LIME examples.

To provide a comprehensive comparison, we also evaluated the interpretability of ECGFounder and ECG-FM alongside our models. On identical ECG examples, DeepECG-SSL demonstrated the most focused attributions, followed by DeepECG-SL, while both ECGFounder and ECG-FM showed diffuse or noisy attribution patterns that were difficult to map to specific clinical features (see Supplementary data online, *Figures S9*–*S12*).

## Discussion

In this study, we introduce two ECG foundation models, DeepECG-SL and DeepECG-SSL, trained using a supervised and self-supervised framework, respectively, on over 1 million ECGs. To demonstrate generalizability, they were validated across 11 geographically diverse external cohorts, including 373 865 public ECGs and 507 538 private ECGs, for a total of 881 403 ECGs, on 4 tasks: multilabel ECG interpretation (77 diagnoses), LVEF prediction, iAF5, and LQTS detection and subtype classification. Both models achieved state-of-the-art performance and maintained AUROC above 0.90 for 76% of diagnoses, despite variation in populations and labelling practices. DeepECG-SSL consistently outperformed DeepECG-SL in low-label settings, particularly LQTS tasks. On the other hand, DeepECG-SL is 60 times smaller and 29 times faster at inference, thereby reducing potential CO₂ emissions by up to 9.7 times on equivalent tasks. A thorough fairness audit stratified by sex and age showed that true-positive rates differed by <0.1 and false-positive rates by <0.02 between groups, indicating near-parity across these categories. Finally, we release a unified preprocessing pipeline with built-in multilingual diagnostic-text extraction, together with all model weights, utilities, and validation code. Making these resources openly available enables inexpensive local fine-tuning and fully reproducible deployment, lowering the barrier to exploring new ECG prediction tasks even when only small, task-specific labelled datasets are at hand while having an edge over competitive models. Our preprocessing pipeline addresses a fundamental challenge in ECG-AI deployment: while experienced clinicians mentally filter equipment-specific artefacts like baseline wander and electrical interference, AI models trained on clean recordings can experience catastrophic performance degradation (AUROC dropping from 0.99 to 0.74) when encountering these artefacts at new sites, making standardized preprocessing essential for real-world generalization.

Recent advances in ECG foundation models include ECGFounder, a supervised algorithm trained on 10 million ECGs, ECG-FM a self-supervised model, and KED^46^ (Knowledge-enhanced ECG) a contrastive learning-based model. All have demonstrated progress in automated ECG interpretation. ECGFounder achieved high internal performance (AUROC ≥0.95 in 82 out of 150 diagnostic labels) but required an order of magnitude more data (11 million ECGs) than our study (over 1 million ECGs). ECG-FM adopts the same WCR-based^14^ SSL training strategy as our model, but it relies on ECG snippets that are 5 s long to predict 14 diagnostic labels. Although this choice effectively doubles the number of training samples, it discards longer temporal patterns that are essential for detecting rhythm disturbances such as second-degree atrioventricular block exemplified by our performance on first-degree atrioventricular block (the closest comparative). DeepECG-SSL has an AUPRC of 0.901 (0.893, 0.908) vs 0.596 (0.58, 0.611) for ECG-FM on CLSA (see Supplementary data online, *Table S40*). Across diagnostic labels overlapping with foundation models, DeepECG-SSL achieved NRI improvements ranging from +0.113 to +1.20, indicating consistently better reclassification of patients into the correct risk groups; however, these comparisons are based on a smaller shared label set intersecting with labels present in the external foundation models. The KED framework used contrastive learning to align ECG signals with ECG text reports. It demonstrated impressive diagnostic performance on single-label classification tasks, with AUROC often exceeding 0.90, without requiring additional training on multiple external datasets; KED^46^ used contrastive learning to align ECG signals with text reports, enabling potentially infinite textual labels. However, it was primarily suited to single-label tasks, may struggle with multilabel scenarios, and did not evaluate or release its model for conditions not explicitly mentioned in ECG reports.

Both DeepECG models qualify as foundation models because they are trained on a large, heterogeneous dataset encompassing a broad range of clinical conditions, contain an extensive number of parameters, and demonstrate strong performance across multiple external datasets. Moreover, they exhibit label-efficient adaptability, making them suitable for various downstream tasks without full re-training. This aligns with the Stanford HAI definition,^47^ which emphasizes comprehensive pre-training on broad data to enable general-purpose capabilities.

Our models demonstrated consistently high AUROC (> 0.95) for common diagnoses such as sinus rhythm, atrial fibrillation, right bundle branch block, and ventricular pacing. Performance remained robust even for more complex labels like Wolff–Parkinson–White, though it varied in rarer conditions (such as Brugada), where limited sample sizes likely contributed to wider confidence intervals. Notably, digital biomarker predictions, such as LVEF and LQTS, traditionally requiring echocardiography or genetic testing, respectively, were accurately derived from routine ECGs, highlighting the potential for earlier, non-invasive detection in clinical practice. Moreover, pre-training provided significantly better results than end-to-end training. For instance, both DeepECG-SL and DeepECG-SSL surpassed Hou *et al*.’s^48^ ECG-based LVEF <50% prediction benchmark, the previous state-of-the-art result, while also delivering lower MAE in LVEF regression. Similarly, both models outperformed our previous iAF5 detector,^33^ with SSL by 0.009 and SL by 0.006 AUROC at the ECG level (*P* < 0.001). Moreover, with a 5 year AF incidence of roughly 5%^49^ and an annual throughput of 100 000 ECGs^50^ at a typical tertiary centre like the MHI, this margin translates to about 75 extra true positives and 1425 additional true negatives. NRI was only marginally higher in the comparator model (0.050 and 0.051, respectively), though this difference was limited to a smaller set of overlapping diagnostic samples. On LQTS detection, Jiang *et al.*^6^ reported slightly higher AUROC values on the MHI-ds test set; however, the 95% confidence intervals overlapped, indicating no meaningful performance difference. Importantly, their model was trained on a dedicated Canadian LQTS registry with 4521 ECGs, nearly twice the number of LQTS waveforms available in our dataset (2741). This substantial data advantage limits direct comparability. With access to their full training cohort, our models would likely achieve even stronger performance.

We addressed four critical barriers to real-world AI adoption: fairness, privacy, resource efficiency, and explainability, linking technical rigour to clinical trust. Fairness evaluations revealed minimal disparities in TPR and FPR across age and sex, mitigating biases against underdiagnosed populations. DeepECG-SSL’s strong performance across diverse centres suggests self-supervised learning can reduce label-related biases. To gauge privacy risks, we used MIA and t-SNE clustering, showing how specialized centres like MHI could be re-identified due to distinct disease distributions. Resource analysis confirmed DeepECG-SL’s suitability for low-resource environments, with faster inference, while DeepECG-SSL’s larger size excelled in data-scarce tasks, even on CPU-based setups. Finally, exploratory LIME^39^ and Grad-CAM^40^ analysis on limited examples showed different attention patterns between models, with more precise attention for SSL than SL, though broader validation is required to assess clinical interpretability. Moreover, aligned with Suh *et al*.^51^ Grad-CAM appears to produce more clinically aligned saliency maps, demonstrating its potential to identify physiologically meaningful ECG waveform regions that correspond to established diagnostic markers. In terms of workflow integration, we envision running these models alongside traditional ECG systems to complement rather than replace existing interpretations. This ‘dual reporting’ approach can bolster diagnostic confidence, refine triage priorities, and ultimately reduce unnecessary downstream testing and is currently being tested in two randomized controlled trials (HEART-AI: NCT06462989^52^ and DAISEA-ECG: NCT06637293^53^) deployed in real time using our DeepECG.ai platform.^54^

Our open-source foundation model targets a critically unmet need by improving diagnostic access in resource-limited settings. Traditional ECG-AI solutions often require large, site-specific labelled data or proprietary software,^55^ which hinders practical deployment in smaller hospitals or under-resourced regions. Our models, combined with our free-text BERT model, have demonstrated that rapid validation is possible across seven EHC, while enabling generalizable results. Moreover, DeepECG’s label-efficient training and robust performance, even for low-data digital biomarker tasks, allow rapid local adaptation with minimal computational overhead. No equivalent ECG-AI foundation model is currently packaged for true plug-and-play, container-based deployment. In contrast, our Dockerized solution is engineered for operational integration: it can expose prediction outputs directly into standard EMR workflows with virtually no additional infrastructure, no custom servers, and no complex orchestration. This dramatically lowers deployment friction, accelerates time-to-value, and positions the model for scalable, enterprise-grade adoption across heterogeneous clinical environments. By enabling earlier detection of conditions like LQTS or low LVEF, often underdiagnosed where advanced cardiac testing is scarce, this approach holds promise for more equitable and cost-effective cardiovascular care worldwide.

Despite promising performance, several critical limitations warrant consideration. While UKB results suggest minimal demographic bias, our predominantly North American validation cohorts necessitate broader evaluation across diverse populations, particularly regarding racial, socioeconomic, and comorbidity dimensions. Also, we acknowledge that several of our large external cohorts primarily include adults aged 45–85, an age range in which age-related cardiovascular abnormalities predominate, so the performance of our models in paediatric populations or early-onset structural or congenital heart diseases remain to be established and will require dedicated validation. The proposed ‘dual-reporting’ approach requires careful examination of clinical workflow integration, including protocols for AI-human discrepancies, alert fatigue mitigation, and liability considerations. Additionally, although we investigated the privacy of our model, we recognize that stronger privacy could be obtained through the utilization of differential privacy;^56^ however, as this approach could lower performance at the cost of privacy, such an approach will incur a privacy-utility trade-off that we did not evaluate in this work. While LIME and Grad-CAM highlight decision drivers in our models, their evaluation on limited selected examples is insufficient to establish that models systematically rely on clinically meaningful waveform features. The clinical relevance and correspondence to established ECG criteria remain unverified across the broader dataset. Comprehensive evaluation with systematic cardiologist review across diverse pathologies and large-scale quantitative assessment would be required before concluding models utilize clinically appropriate decision-making pathways. Label variability in clinical datasets remains a concern, though robust external performance partially mitigates this issue. Moreover, we did not directly compare model performance with an expert committee, but our reported accuracy aligns with or surpasses that of prior AI studies tested against cardiologist-level performance.^3^ Our framework’s demonstrated efficiency, achieving AUROC 0.97 with just 100 000 ECGs, suggesting sufficient performance with modest dataset sizes, contrasting with more data-intensive domains. Future work will prioritize prospective validation studies, systematic failure mode analysis (particularly for rare pathologies), exploration of multimodal data integration, while maintaining focus on real-world clinical utility and dedicated investigation into the origin and mitigation of wave artefacts observed in MHI-ds ECG signals. Lastly, we did not adjust *P*-values for multiple comparisons, so results should be interpreted with caution in the context of multiple testing.

We present two extensively validated ECG foundation models across multiple clinical settings. DeepECG-SSL excels in emerging biomarker detection with limited training data (including LQTS subtyping and LVEF classification), while the more efficient DeepECG-SL matches performance on traditional diagnoses despite being substantially smaller. Both models demonstrate robust external generalization across most diagnostic categories, minimal demographic bias, and clinically aligned feature detection. Our open-source pipeline enables reproducible deployment and efficient local adaptation through standardized preprocessing and Dockerized implementation. Two ongoing randomized trials evaluate clinical integration alongside existing ECG systems, with future work focused on expert committee validation and expanded clinical applications. These contributions establish a comprehensive framework for responsible, clinically validated AI in cardiovascular diagnostics.

## Supplementary Material

ehaf1119_Supplementary_Data

## Declarations

### Data Availability

The internal MHI dataset and external private datasets from health centres UW, UCSF, MGH, JGH, CSH, and NYP are not publicly available due to its potentially identifiable nature of patients in these datasets. However, MIMIC-IV is accessible from https://physionet.org/content/mimiciv/3.1/, UKB is available upon request from https://www.ukbiobank.ac.uk/enable-your-research/apply-for-access, Code-15 can be accessed from https://zenodo.org/records/4916206, PTB is available from https://physionet.org/content/ptb-xl/1.0.3/, and CLSA is available upon request from https://www.clsa-elcv.ca/data-access/.

The code for data preprocessing, model training, and fine-tuning is available on the GitHub page: https://github.com/HeartWise-AI/DeepECG_Docker/tree/main. The model weights can also be accessed from the same page.
