# Correction to: Foundation models for electrocardiogram interpretation: clinical implications

**DOI:** 10.1093/eurheartj/ehag139

**Published:** 2026-03-13

This is a correction to: Alexis Nolin-Lapalme, Achille Sowa, Jacques Delfrate, Olivier Tastet, Denis Corbin, Merve Kulbay, Derman Ozdemir, Marie-Jeanne Noël, François-Christophe Marois-Blanchet, François Harvey, Surbhi Sharma, Minhaj Ansari, I Min Chiu, Valentina D’souza, Sam F Friedman, Michaël Chassé, Brian J Potter, Jonathan Afilalo, Pierre Adil Elias, Gilbert Jabbour, Mourad Bahani, Marie-Pierre Dubé, Patrick M Boyle, Neal A Chatterjee, Joshua Barrios, Geoffrey H Tison, David Ouyang, Mahnaz Maddah, Shaan Khurshid, Julia Cadrin-Tourigny, Rafik Tadros, Julie Hussin, Robert Avram, Foundation models for electrocardiogram interpretation: clinical implications, *European Heart Journal*, 2026; https://doi.org/10.1093/eurheartj/ehaf1119

The following change has been made to the originally published paper. Text in the **Acknowledgement** section has been emended to read: “We further acknowledge that this research has been conducted using Comprehensive Baseline and Follow-up 12-lead ECG data and Raw Data under Application ID 2401002 from the CLSA dataset: Comprehensive Follow-u= 2 v2.0, Baseline, and Follow-up 1 ECG Images and Raw Data, under Applicat=on ID 2401002.” instead of: “We further acknowledge that this research has been conducted using Comprehensive Baseline and Follow-up 12-lead ECG data and Raw Data under Application ID 2401002 from the CLSA dataset.”

